# Major Flame Burn Caused by Electric Fly-Swatter

**Published:** 2007-02-05

**Authors:** P. Muangman, J. R. Scott, K. Keorochana

**Affiliations:** a Burn Unit, Trauma Division, Department of Surgery, Faculty of Medicine, Siriraj Hospital, Mahidol University, Bangkok, Thailand; b Division of Plastic Surgery, Harborview Medical Center, University of Washington, Seattle

In Thailand and in many parts of the world, the annoyances and potential health hazards of mosquitoes^1^ have led to the development of many devices designed to kill them.^2^ The electric fly-swatter (Yongtong,Yongtong Electronics Co, Ltd, China) is thought by many to be a safe antimosquito device that is used widely for killing mosquitoes in tropical countries (Figure 1). It is free of toxic chemicals or other poisonous materials, and it combines the common household fly-swatter with an electrical screen designed to incinerate the insect.^3^ Unfortunately, the tennis racket shape, the sparkling sound of electrical shock, and a flash of light with incineration of the fly can be enticing to children.

A 2-year-old boy was admitted to the Siriraj Burn Unit, Siriraj Hospital, Bangkok, Thailand, following a 25% body surface area burn. The boy entered a workroom in the home where his father was painting flammable adhesive shoe glue on the floor (Elephant Adhesive Glue, TN Chemical Supply, Bangkok, Thailand). He sat down near his father and soon began waving the electric fly-swatter device toward a mosquito. After the insect made contact with the metallic mesh screen, an electrical arc was generated and the insect was incinerated. The boy then placed the hot device onto the floor, which ignited the glue and, in turn, the boy's clothing. The boy's physical examination on his arrival revealed superficial partial-thickness burns to his face, neck, left upper extremity, posterior trunk, and bilateral lower extremities (Figure 2). The burn wound was immediately cleansed and debrided and dressed with 1% silver zinc sulfadiazine cream daily until the wounds had healed. The patient was discharged from the hospital after 24 days. No infection or other complications were observed during the hospital stay.

Although the electric fly-swatter is a useful personal insect eliminator, care must be taken to prevent application of the metallic mesh to flammable surfaces and to allow sufficient time for cooling of the mesh following its use.^3^ Adhesive shoe glue is a known flammable substance with the disastrous ability to ignite spontaneously upon contact with heat. It should never be used in close proximity to electrical equipment, sources of static electricity, or machinery with moving parts, and it should be stored in closed airtight safety containers after use. We also recommend a heightened awareness of the risks associated with the use of the electric mosquito swatter, which should be kept out of children' reach. Guidelines for safe handling of the device are in place.

## Figures and Tables

**Figure 1:**
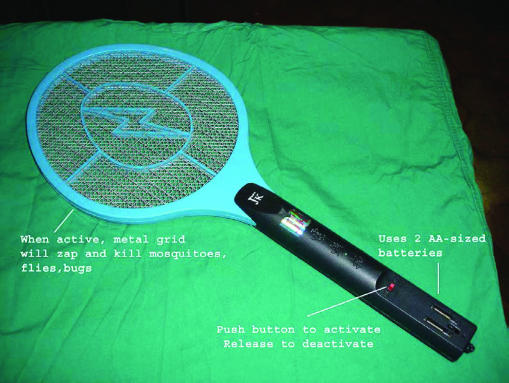
The electrical fly-swatter.

**Figure 2:**
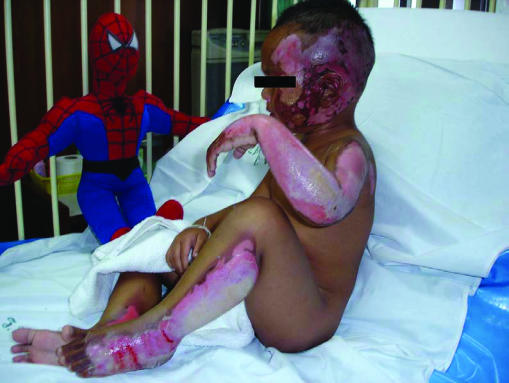
The appearance of the patient revealed second degree 25% total body surface area burn.

## References

[1] Guerra CA, Snow RW, Hay SI (2006). A global assessment of closed forests, deforestation and malaria risk. Ann Trop Med Parasitol.

[2] Okabayashi H, Thongthien P, Singhasyanon P (2006). Keys to success for a school-based malaria control program in primary schools in Thailand. Parasitol Int.

[3] Pollick M What is an electric fly-swatter?. http://www.wisegeek.com/what-is-an-electric-fly-swatter.htm.

